# Zein nanoparticles as oral carrier for mometasone furoate delivery

**DOI:** 10.1007/s13346-023-01367-y

**Published:** 2023-05-19

**Authors:** Priscila Zimath, Soraia Pinto, Sofia Dias, Alex Rafacho, Bruno Sarmento

**Affiliations:** 1 Laboratory of Investigation in Chronic Diseases, Department of Physiological Sciences, Center of Biological Sciences, Federal University of Santa Catarina, Florianópolis, Brazil; 2 Graduate Program in Pharmacology, Center of Biological Sciences, Federal University of Santa Catarina, Florianópolis, Brazil; 3 i3S - Instituto de Investigação e Inovação em Saúde, University of Porto Rua Alfredo Allen, 208 | 4200-135 Porto, Portugal; 4 ICBAS, Instituto de Ciências Biomédicas Abel Salazar, University of Porto, Porto, Portugal; 5 IUCS - CESPU, Gandra, Portugal

**Keywords:** Zein, Mometasone furoate, Glucocorticoid, Nanoparticles, Mucus-permeating, Oral delivery

## Abstract

Mometasone furoate (MF) is a synthetic glucocorticoid used clinically to treat specific inflammatory disorders including superior and inferior respiratory tract. Due to its poor bioavailability we further investigated whether nanoparticles (NPs) made of zein protein may constitute a safe and effective choice to incorporate MF. Thus, in this work, we loaded MF into zein NPs aiming to evaluate possible advantages that could result from oral delivery and extend the range of MF application such as inflammatory gut diseases. MF-loaded zein NPs presented an average size in the range of 100 and 135 nm, narrow size distribution (polydispersity index < 0.300), zeta potential of around + 10 mV and association efficiency of MF over 70%. Transmission electron microscopy imaging revealed that NPs had a round shape and presented a smooth surface. The zein NPs showed low MF release in a buffer that mimics the gastric condition (pH = 1.2) and slower and controlled MF release in the intestinal condition (pH = 6.8). The short and intermediate safety of zein NPs was confirmed assessing the incubation against Caco-2 and HT29-MTX intestinal cells up to 24 h. Permeability studies of MF across Caco-2/HT29-MTX co-culture monolayer evidenced that zein NPs modulated MF transport across cell monolayer resulting in a stronger and prolonged interaction with mucus, potentially extending the time of absorption and overall local and systemic bioavailability. Overall, zein NPs showed to be suitable to carry MF to the intestine and future studies can be developed to investigate the use of MF-loaded zein NPs to treat intestinal inflammatory diseases.

**Graphical abstract:**

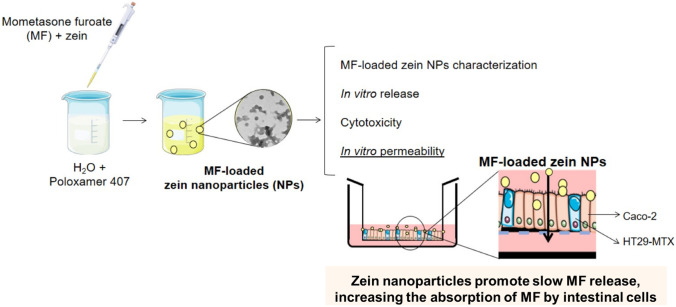

**Supplementary Information:**

The online version contains supplementary material available at 10.1007/s13346-023-01367-y.

## Introduction

Exogenous glucocorticoids (GCs) are broadly used in different disorders as anti-inflammatory and immunosuppressive agents. But although their therapeutic effects, the use of GCs remains limited and presents low patient adherence since the administration of these drugs can cause severe side effects, particularly when used in high doses and for extended periods [1, 2]. To reduce these complications, it was developed a new generation of GCs that allowed to enhance their therapeutic potency and accelerate the clearance pharmacokinetic property of this class of drugs [3], which included fluticasone propionate [4] and mometasone furoate (MF) [5]. Both drugs are generally delivered by intranasal or inhaled routes, and they are employed as first-line treatment in different pathologies that affect the respiratory tract.

MF is a highly lipophilic compound with poor aqueous solubility developed for local administration and when administered by inhalation reveals low bioavailability, in which usually only less than 1% of the administered drug reaches the systemic circulation [6]. Moreover, a human pharmacokinetic study demonstrated that the half-life of MF following the intravenous route was 4.45 h, and MF was still detected in the bloodstream up to 8 h after administration [6]. The low availability that MF presents might be justified by its lipophilic character and the various obstacles, hindering the drug accessibility to the absorptive epithelium. Although MF is not available in the clinic in oral formulations, previous studies in rodents demonstrated that MF by oral administration, either for acute or prolonged periods, demonstrated to maintain anti-inflammatory activity while concurrently reducing the side effects normally associated with the use of this drug [7, 8]. In a previous study, our group showed that MF treatment prevents acute peritonitis in both male and female rats and also attenuates chronic colitis in mice [7]. While subchronic oral administration of MF results in reduced insulin sensitivity it does not affect either glucose tolerance or plasmatic lipid profile in male and female rats, as dexamethasone treatment does at the same dose [7]. Considering oral MF treatment preserves anti-inflammatory properties with minor diabetogenic effects, this opened the possibility to study a new formulation to carry MF to the intestine, to be further investigated in a broader range of intestinal inflammatory diseases.

Nanotechnology-based drug systems hold enormous and promising potential in the oral delivery field since this technology can be used as a useful strategy for the (i) delivery of poorly water-soluble drugs, (ii) targeting of drugs to a specific part of the gastrointestinal tract, and (iii) transcytosis of drugs across the tight intestinal barrier [9, 10]. Besides, nanotechnology can be applied to help to overcome several adverse effects and formulation challenges, that are usually related to GCs [11–13]. For instance, the development of nanoparticles (NPs)-loading GCs can allow to obtain minimal adverse effects and a more convenient route of administration, leading to improved patient compliance and enhanced therapeutic effect [14–16]. For this purpose, zein has been extensively used in the pharmaceutical industry to develop NPs suitable to use as carrier systems, taking advantage of its assets, since it is a natural corn protein that presents desirable characteristics as safety, biodegradability, and low toxicity [17–26]. Zein is hydrophobic in nature material in which its matrix is constituted mainly by lipophilic amino acids residues (> 50%), which confers it the capacity to attract and encapsulate hydrophobic compounds [27]. Additionally, previous studies described the capacity of zein NPs to improve the oral bioavailability of compounds or offer a slow release of both small [21, 23, 24, 26, 28] and large molecules [21]. Moreover, the mucoadhesive properties associated with zein NPs provide prolonged residence in close contact with the gut mucosa [23]. Considering that previous studies have demonstrated that MF by oral delivery presents fewer adverse effects [7, 8, 29], our goal was to produce and characterize zein NPs loaded with MF to develop a novel strategy for orally administered MF, to enhance the local concentration of the drug in the intestine that could be of clinical relevance when considering inflammatory-related gut diseases. We also investigated the permeability of MF-loaded zein NPs in the intestinal co-culture in vitro model.

## Materials and methods

### Materials

Ethanol (absolute degree), hydrochloric acid (HCl), sodium chloride (NaCl), monopotassium phosphate (KH_2_PO_4_), and sodium hydroxide (NaOH) are common reagents supply. Zein (38 kDa, content in protein 81.9–100%, calculated on a dry basis) and triazolyl blue tetrazolium bromide (MTT) were acquired from Sigma Aldrich (Sto. Louis, MO, USA). Mometasone furoate was kindly provided by Ache Pharmaceuticals (Brazil). Poloxamer P407 (Kolliphor^®^ P 407) was kindly provided by BASF (BTCEurope, Barcelona, Spain). Dulbecco’s Modified Eagle’s Medium (DMEM) and Hanks’ Balanced Salt solution (HBSS) were purchased from Gibco (Waltham, USA). Fetal bovine serum (FBS) was purchased from Biochrom AG (Berlin, Germany). The non-essential amino acid was purchased from Gibco (Paisley, UK). Amicon Ultra filter 100 kDa (Ultracel membrane with 100,000 MWCO, Millipore Corporation, Bedford, MA, USA). Polyethylene terephthalate membrane inserts were purchased from Millicel^®^ Merck (Darmstadt, Germany). Ultrapure water was obtained using a Milli-Q purification system (Merck Millipore). All other solvents and chemicals were of analytical grade or equivalent.

## Production of MF-loaded zein NPs

MF-loaded zein NPs were produced by the nanoprecipitation technique [26]. Briefly, MF was dissolved in 1 mL of ethanol and added to 2 mL of a 70% (v/v) ethanol solution containing zein (10 mg/mL). This solution was magnetically stirred over 1 h at room temperature (RT) (300 rpm). Further, it was added dropwise to 10 mL of 0.1 or 0.5% (w/v) Poloxamer 407 solution under magnetic stirring (200 rpm), using a 1 mL micropipette. NPs were collected after 3 h of stirring at RT and centrifuged for 40 min through a filter device (molecular weight cutoff of 100 kDa) at 2500 × g. Different MF theoretical loadings (TL) (5% and 10%) and Poloxamer 407 concentrations (0.1 and 0.5% (w/v)) were produced, namely 5% of MF TL and 0.1% (w/v) of Poloxamer 407 (5% MF 0.1P), 5% of MF TL and 0.5% (w/v) of Poloxamer 407 (5% MF 0.5P), and 10% of TL and 0.5% (w/v) of Poloxamer 407 (10% MF 0.5P).

## Physical–chemical characterization of zein nanoparticles

### Particle size, polydispersity index, and zeta potential

Empty and MF-loaded NPs were dispersed in 10 mM NaCl solution (0.2 mg/mL) adjusted at pH 4.0 with HCl 0.5 M and characterized regarding average size and polydispersity index (PdI) by dynamic light scattering (DLS), and zeta potential (ZP) by laser Doppler electrophoresis using a Zetasizer Nano ZS (Malvern Instruments, Malvern, UK). All measurements were performed in triplicate at 25 °C.

## Stability of MF-loaded zein NPs

Liquid suspensions of empty and MF-loaded zein NPs were stored at 4 °C for 30 days after production. The average particle size, PdI, and ZP were evaluated as described above.

## Surface morphology

Transmission electron microscopy (TEM, Zeiss model EM 902 A) was used to analyze the shape of fresh NPs. For TEM analysis, samples were deposited on metal stubs with carbon tape and sputter-coated with a thin layer of gold/palladium using the SPI Module Sputter Coater equipment (Structure Probe, Inc., West Chester, PA, USA).

## MF association efficiency and drug loading

After the production of zein NPs, the association efficiency (AE) and drug loading (DL) of MF were determined by the direct method. Briefly, 1 mL of fresh zein NP suspensions were placed into semi-stoppered glass vials and frozen at − 80 °C for 12 h. Samples were then relocated to a Modulyo 4 K freeze dryer (Edwards, Crawley, UK) at 0.09 mbar and freeze-dried for 24 h. Next, lyophilization was done at a condenser surface temperature of − 50 ± 5 °C. After lyophilization, NPs were reconstituted in 1 mL of dimethyl sulfoxide (DMSO) to destroy the particles. Samples were left under static conditions for 2 h at RT, and then, each solution was measured using an ultraviolet-visible (UV-Vis) spectrophotometer as follow described [30].

The AE (%) and DL (%) of the MF-loaded zein NPs were quantified using the following equations:

1 $$\text{AE}\; \left(\%\right)=\frac{\mathrm{Amount \;of\; MF}\,-\,\mathrm{loaded \;to\; NPs}}{\mathrm{The\; total \;amount \;of \;MF}}\times100$$

2 $$\text{DL}\; \left(\%\right)=\frac{\mathrm{Amount \;of\; MF}\,-\,\mathrm{loaded \;to \;NPs}}{\mathrm{The \;total \;amount \;of \;MF }\,+\,\mathrm{ Amount \;of \;zein}}\times 100$$

## MF quantification and method validation

Perkin Elmer Lambda 25 UV/Visible Spectrophotometer with 10 mm matched quartz cells was used. To prepare the calibration curve, firstly, a standard stock solution containing 1000 μg/mL of pure MF was prepared in ethanol. Further, appropriate dilutions done in the range of MF concentrations from 5 to 60 μg/mL were scanned and absorbances were measured at selected wavelengths (wavelength range from 200 to 300 nm), as previously described [30]. Calibration curves to test linearity were obtained by plotting absorbance (Abs) and concentration of MF (μg/mL), as shown in Suppl. Fig. 1A, B. Precision was estimated by inter-day analysis, repeating the quantification of different samples (40 μg/mL MF) on three consecutive days in triplicate. Results were expressed as % of drug and relative standard deviation (%, RSD) in Suppl. Fig. 1C.

## In vitro drug release of MF-loaded NPs

The in vitro release of MF from zein NPs was evaluated in buffers that mimic the gastric (8% (v/v) 1 M of HCl, 0.2% (w/v) of NaCl at pH 1.2 and 1.5% (w/v) of Poloxamer 407) and intestinal (0.68% (w/v) of KH_2_PO_4_, 7.7% (v/v) of 0.2 N NaOH and 1.5% (w/v) of Poloxamer 407 at pH 6.8) environment. The experiment was performed over 8 h at 37 °C and 100 rpm in an incubator shaker (IKA KS 4000 control, IKA^®^-Werke GmbH & Co. KG, Staufen, Germany).

Briefly, 1 mL of NPs (corresponding to 1.0 or 2.0 mg of MF) was placed on a dialysis membrane (Snakeskin dialysis tubing, 10000 MWCO, and 22 mm diameter) and further emerged in a glass container with 14 mL of buffer that mimic gastric condition. Then, aliquots of 0.2 mL were collected at different time points (15, 30, 45, 60, 90, and 120 min), and the same volume was replaced with pre-heated buffer. After 2 h of the experiment at acidic pH, the dialysis membrane was transferred to a glass container with 14 mL of buffer that mimics intestinal condition. Aliquots of 0.2 mL were also collected at different time points (150, 180, 240, 360, and 480 min), and the same volume was replaced with pre-heated intestinal fluid.1 The amount of MF released from zein-NPs over time was quantified by UV–Vis. The experiment was performed in sink conditions.

## Cell culture

### Cell lines and culture conditions

Human colonic epithelial Caco-2 cells were obtained from the American Type Culture Collection (ATCC, Manassas, VA, USA) and used in passages 30–35. Human colonic epithelial HT29-MTX cells were kindly provided by Dr. T. Lesuffleur (INSERM U178, Villejuif, France) and used in passages 45–55. The cell lines were maintained in DMEM with ultraglutamine supplemented with 10% (v/v) of fetal bovine serum heat inactivated, 100 U/mL of penicillin and 100 μg/mL of streptomycin, and 1% (v/v) of non-essential amino acids.

## Cytotoxicity of MF-loaded zein NPs

The effect of MF-loaded zein NPs in the viability of Caco-2 and HT29-MTX colorectal cell lines was assessed using the MTT metabolic activity assay. Briefly, Caco-2 or HT29-MTX cells were seeded in 96-well plates at the cellular densities of 20.000 and 10.000 cells/well, respectively, and incubated for 24 h at 37 °C and 5% CO_2_. After, the medium was removed, the wells were washed twice with pre-warmed phosphate buffered saline solution (PBS) at pH 7.4, and the samples suspended in DMEM were added to the wells. MF-loaded zein NPs and free MF treatments were assessed at the following MF concentrations: 0.1, 1, 10, 30, 50, and 100 µM. Regarding the free MF solutions tested, a stock solution of MF at 1000 M in DMSO was previously prepared and then diluted in DMEM. Cells were also incubated in the presence of DMEM containing 0.5% (v/v) of DMSO to assess the toxicity of the organic solvent used to prepare MF solutions. The positive control of 100% of viable cells was obtained by incubating the cells with DMEM, while the negative control, equivalent to cell death, was acquired by treating cells with 1% (v/v) of Triton^®^ X-100 in DMEM. Both intestinal cell lines were incubated with the samples for 4 h or 24 h at 37 °C and 5% CO_2_. Then, the wells were washed twice with pre-warmed PBS at pH 7.4 to remove the samples, and 200 µL of the MTT reagent (0.5 mg/mL in PBS) was added to each well and incubated for 4 h. Subsequently, the content of the wells was removed, 200 µL of DMSO was added, and the plate was stirred at 100 rpm for 20 min to solubilize the formazan crystals originating from metabolic active viable cells. The absorbance was measured at 570 nm (test wavelength) and 630 nm (background wavelength) using a microplate reader Synergy 2^®^ (Biotek Instruments). Tests were performed in three independent experiments, each performed in triplicate. The following equation was used to determine the cell viability (%):

3 $$\mathrm{Cell\; viability\;(\mathrm{\%})}=\frac{(\mathrm{experimental \;value }\,-\,\mathrm{ negative \;control})}{(\mathrm{positive\; control }\,-\,\mathrm{ negative\; control})}\times \,100$$

## In vitro permeability

For the in vitro permeability experiment, caco-2 (passages 35–40) and HT29-MTX (passages 45–50) cells were seeded in a ratio of 9:1 at a final density of 1 × 10^5^ cells.cm^−2^ on the apical compartment of the Transwell^®^ insert (0.5 mL) as previously described [26, 31, 32]. Moreover, 1.5 mL of DMEM was added to the basolateral compartment. Then, the cells were maintained in culture for 21 days and the medium was replaced from both compartments every two days. The evolution of growth and the epithelial integrity of co-culture monolayers was evaluated by the transepithelial electrical resistance (TEER) measurement, using the EVOM^2^ epithelial volt ohmmeter equipped with an STX2 electrode (World Precision Instruments, Inc., Sarasota, FL, USA) before medium replacement. After 21 days, DMEM was removed from apical and basolateral compartments and both sides of the inserts were carefully washed with pre-warmed (37 ºC) PBS. Then, the medium was replaced by pre-warmed HBSS, containing 0.2% (w/v) of Poloxamer 407 to guarantee sink conditions and allowed to equilibrate for 30 min at 37 °C. Further, the HBSS of the apical compartment was replaced by 0.5 mL of free MF (50 μM, dissolved in a final concentration of 0.1% of DMSO), or 0.5 mL of MF-loaded zein NPs (5% MF 0.1P, 5% MF 0.5P or 10% MF 0.5P) equivalent to 50 μM of MF suspended in HBSS with 0.2% (w/v) of Poloxamer 407. The plates were located in an orbital shaker incubator at 100 rpm and 37 °C. Aliquots of 200 µL were collected from the basolateral compartment at several time points (15, 30, 60, 120, and 240 min) and replaced for the same volume of pre-warmed HBSS. After the last time point, the medium was removed from both compartments of the inserts, and cells were washed twice with HBSS at 37 °C. The TEER parameter was measured before the experiment and at each time point to ensure the monolayer integrity during the whole assay. At the end of the experiment, the membrane was detached from the insert and the cell monolayer was lysed with DMSO for further quantification. The amount of MF that permeated the intestinal monolayer over time was quantified by UV–Vis spectroscopy [33]. Apparent permeability (Papp) values were calculated according to the equation:

4 $$Papp=\frac{dQ \,(A\,\times \,C0)}{dt}$$

where *dQ* is the amount of MF that permeated the monolayer, *A* is the diffusion area (cm^2^), *C0* is the initial concentration of MF (μg cm^3^), and *dt* is the total time of experiments. The coefficient *dQ/dt* represents the steady-state flux of MF across the monolayer.

## Statistical analysis

All analyses were performed using GraphPad Prism Version 8.01 software (GraphPad Inc., La Jolla, CA, United States). Data were primarily tested for normal distribution and homogeneity test of variance. Grubb’s test was applied to determine whether any value had reached a significant outlier, limited to 10% of samples. For scatter plot with bar graphs, one-way ANOVA was applied with Tukey’s post hoc test or Kruskal–Wallis with Dunn’s post hoc test. For stability of MF-loaded zein NPs unpaired t-test was applied. Two-way ANOVA followed by Dunnett’s multiple comparison test was used for the cumulative MF release from NPs formulations and for the in vitro cumulative permeability analysis. The results were expressed as mean ± SD for parametric data or as median and interquartile ranges for non-parametric data. Significance was set at *p* < 0.05.

## Results and discussion

### Characterization of MF-loaded NPs

There is a growing interest in the use of protein-based nanocarriers as a drug delivery system, as they are biodegradable, easily obtained, and with high drug- and cell-binding capacity, essential features in drug delivery applications [34]. Thus far, zein protein has been exploited and considered a good candidate for use in food and pharmaceutical industries for different applications, including for the oral delivery of proteins [21] and vegetable-derived materials [35]. By taking advantage of its properties and because zein NPs constitute a promising carrier for hydrophobic compounds like MF, we aim to produce MF-loaded zein NPs by the nanoprecipitation methodology.

NPs characterization is of the utmost relevance since parameters like particle size and size distribution can influence the drug loading, release, toxicity, in vivo distribution, and particle stability [36]. Therefore, different MF theoretical loadings (TL) (5% and 10%) and Poloxamer 407 concentrations (0.1 and 0.5% (w/v)) were tested. The empty zein NPs, 0% of MF TL and 0.5% (w/v) of Poloxamer 407 (0% MF 0.5% P), and the MF-loaded zein NPs (5% MF 0.1P, 5% MF 0.5P and 10% MF 0.5P) were physicochemical characterized regarding their average size, PdI, ZP, AE and DL (Table 1). Empty zein NPs (0% MF 0.5P) and MF-loaded zein NPs presented an average size of 118 nm and between 100 and 140 nm, respectively. Particularly, when comparing 5% MF 0.5P and 10% MF 0.5P formulations, the average size of MF-loaded zein NPs increased with the increase of MF TL, fact that was also observed for the PdI values presented in Table 1. These results may be justified by the association of higher amounts of MF molecules within the zein matrix, causing an increase in the NP size. In fact, it has been reported that the size of NPs plays an important role and can influence the oral bioavailability, in which smaller-sized particles (50 and 200 nm in diameter) can be absorbed and cross the intestinal epithelial layer in a more effective way, when compared with larger-sized particles [37]. Actually, this size range is reported to allow a better internalization by enterocytes and M cells and subsequently, a better transport across the gastrointestinal tract [38].

**Table 1:** Average size (nm), polydispersity index (PdI), zeta potential (ZP, mV), association efficiency (AE, %), and drug loading (DL, %) values of MF-loaded NPs at different theoretical drug loadings. Results are presented as mean ± SD (n = 3)

**NPs**	**Size (nm)**	**PdI**	**ZP (mV)**		
				**AE (%)**	**DL (%)**
**0% MF 0.5P**	118 ± 20	0.130 ± 0.020	12.5 ± 1.5	**-**	**-**
**5% MF 0.1P**	108 ± 10	0.254 ± 0.020	10.6 ± 2.2	77 ± 7	3.7 ± 0.3
**5% MF 0.5P**	102 ± 8	0.221 ± 0.040	8.7 ± 2.3	97 ± 2	4.6 ± 0.1
**10% MF 0.5P**	131 ± 6	0.314 ± 0.050	7.4 ± 1.2	71 ± 12	6.5 ± 1.4

Additionally, the values of PdI for each formulation are equal to or lower than 0.300, indicating the homogenous size distribution of the NPs [39]. The ZP values of the formulations were around + 7 and + 10 mV, surface charges that were already expected accordingly to the literature for either zein NPs produced without surfactant [26], or when using poloxamer as a surfactant [25, 40] and for pure zein [39]. Indeed, a positive surface charge is reported to be an important factor that is related with NPs adsorption onto the cell membranes, improving NP uptake by direct permeation and consequent effectiveness, compared to neutral and negatively charged NPs [41]. In addition, it was anticipated that the zein NPs would present a positive surface charge since the dispersant media used for particle measurement presents a pH of 5.5, lower than the isoelectric point of the zein protein (pH 6.2) [42]. Regarding AE, a higher value of MF (> 90%) was obtained for the 5% MF 0.5P formulation comparatively with the 5% MF 0.1 P (AE = 77 ± 7%) and 10% MF 0.5 P (AE = 71 ± 12%) formulations, as described in Table 1. Overall, the 10% MF 0.5P formulation had the highest average size and PdI, and the lowest AE comparatively with the other formulations, which can possibly be explained due to the saturation solubility of MF in the solvent that might affect the size distribution of the NPs. And when comparing the characterization results described in Table 1 obtained for the empty and MF-loaded NPs, it indicates an effective entrapment of the drug within the polymeric matrix (0.5% Poloxamer 407). Previous studies that involved the production of small NPs with poloxamer and lecithin in the same formulation demonstrated a positive correlation between the amount of zein used to produce the NPs and their mean diameters; contrarily, the emulsifier concentrations and the NPs mean diameters demonstrated to be inversely proportional [43, 44]. The addition of surfactant poloxamer during the preparation of the zein NPs allowed to reduce the hydrophobic attraction of the lipophilic residues and increase colloidal repulsion, properly establishing a stable nanocomposite system [19].

**Fig. 1:**
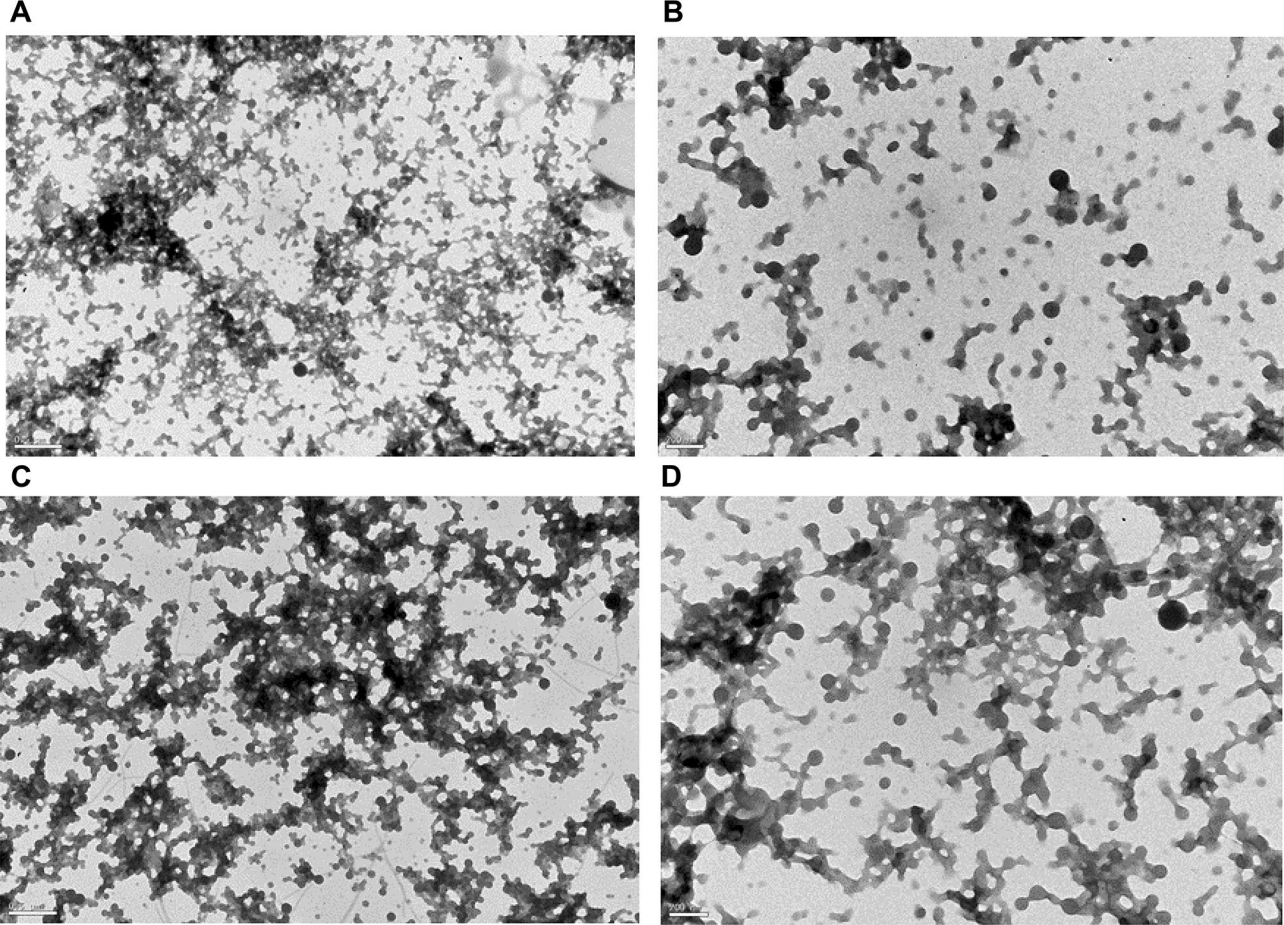
Representative transmission electron microscopy (TEM) images of unloaded zein nanoparticles (NPs) (**A** and **B**) and MF-loaded zein NPs with 5% (**C** and **D**) theoretical DL and 0.5% Poloxamer 407 (5% MF 0.5P). Scale bar represents 500 nm (**A** and **C**) and 200 nm (**B** and **D**)

The morphology of the zein NPs was evaluated by TEM, showing a round shape and smooth surface (Fig. 1), properties that contribute to an effective drug delivery [36]. Moreover, TEM images confirmed size and PdI results of zein NPs. The association of increasing amounts of 5% of MF (5% MF 0.5P NPs) had no apparent impact on the morphology of zein NPs (Fig. 1C, D).

**Fig. 2:**
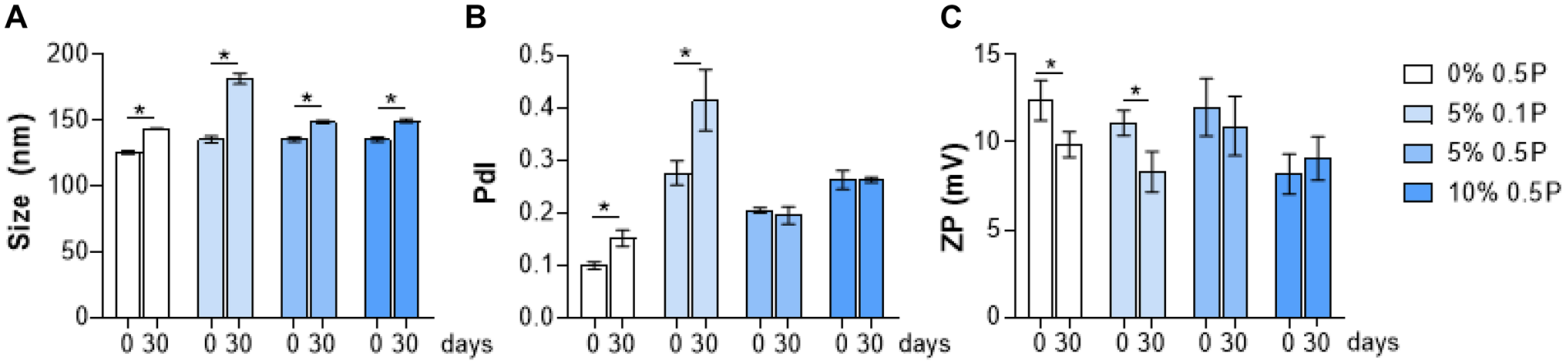
Characteristics of fresh mometasone furoate (MF)-loaded zein nanoparticles (NPs) (day 0), and after 30 days stored at 4 °C. Size expressed as hydrodynamic diameter (nm) (**A**), polydispersity index (PdI) (**B**), zeta potential (ZP, mV) (**C**) of unloaded zein NPs (0% o.5P) and 5% or 10% MF-loaded zein NPs DL containing 0.1% (5% 0.1P) or 0.5% of Poloxamer 407 (5% 0.5P 10% 0.5P). Results were expressed as mean ± SD values (n = 3). Unpaired t-test was used for statistical analysis. The significant differences were set at probabilities of *p < 0.05

Zein NPs have been acknowledged as reliable systems for drug delivery applications, permitting the development of nanoformulations that can enhance the properties of the encapsulated compounds to increase their effective delivery. Hence, the optimization of the zein NPs obtaining method is urgently needed to enhance its chemical stability, so it allows to overcome formulation challenges like aggregation and precipitation of the zein NPs [45]. In this context, strategies like coating with emulsifiers such as carrageenan, arabic gum, lecithin, poloxamer, sodium caseinate, pectin, and chitosan, are being employed and considered to maintain repulsion among the particles, leading to the successful stability improvement of the zein NPs [46, 47]. To analyze the stability of zein NPs, liquid suspensions of NPs were stored at 4 °C for 30 days, followed by the assessment regarding size, PdI, and ZP(Fig. 2A–C). After 30 days of storage of the NPs at 4 °C, an increase in the average size was observed for all formulations (Fig. 2A), being more noticeable in the 5% MF 0.1P formulation. The PdI values were similar after 30 days of the experiment, except for the 5% MF 0.1P formulation, in which the PdI value increased (Fig. 2B). The ZP values decreased in empty NP and 5% MF 0.1P (Fig. 2C). Thus, formulation enhancements will be necessary to maintain the stability of the zein NPs. Overall, the MF-loaded NPs, 5% MF 0.5P was the formulation with the greatest homogenous distribution and highest AE, retaining a suitable amount of drug inside the particles (DL of around 5%).

## In vitro drug release of MF-loaded NPs

**Fig. 3:**
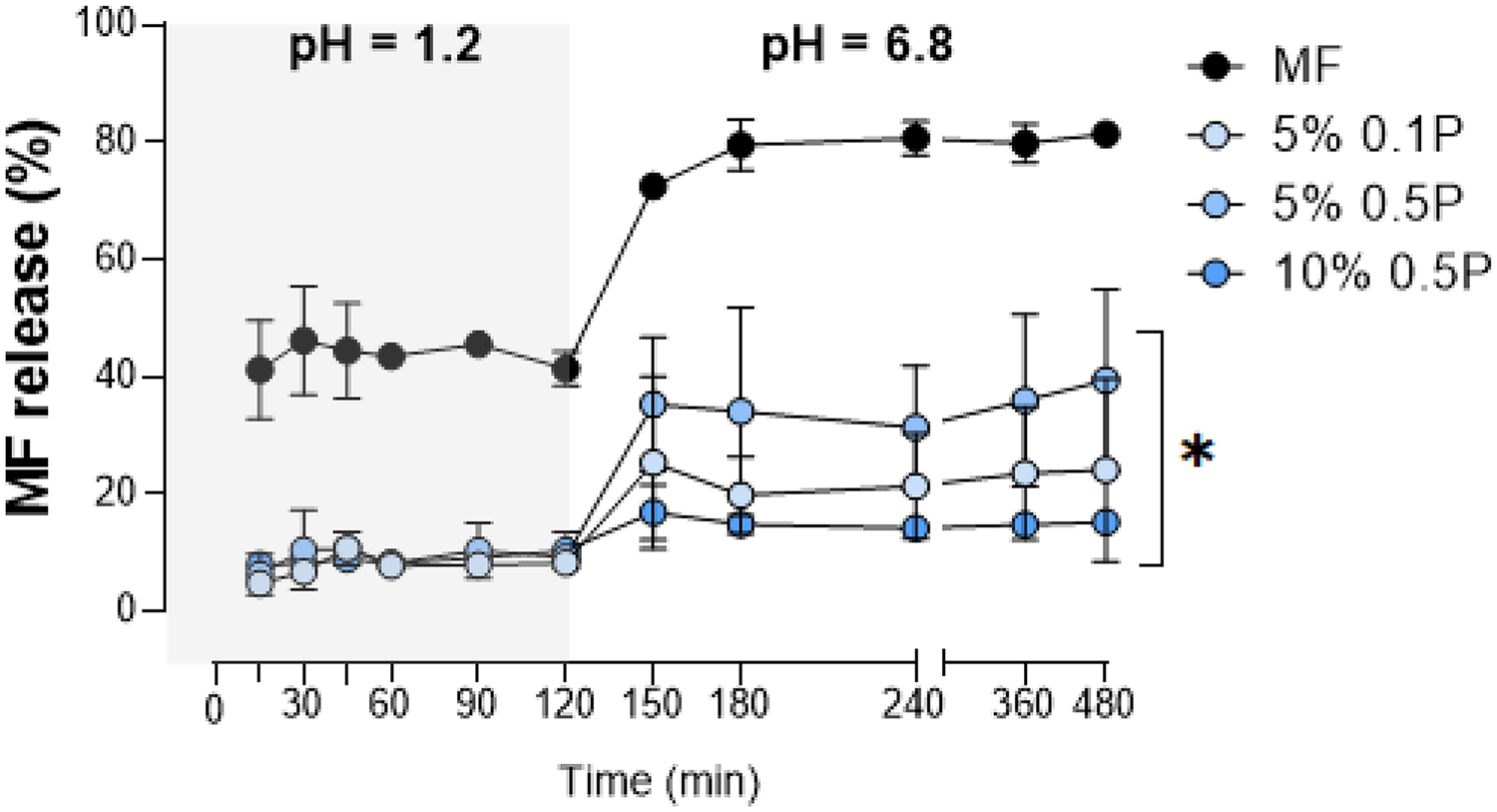
Cumulative mometasone furoate (MF) release from zein nanoparticles (NPs) at different theoretical loadings and different concentrations of Poloxamer 407: 5% or 10% MF-loaded zein NPs containing 0.1% (5% 0.1P) or 0.5% of Poloxamer 407 (5% 0.5P 10% 0.5P). Results are expressed as mean ± SD (n = 3). Two-way ANOVA followed by Dunnett’s multiple comparison test was used for statistical analysis. The significant differences were set at probabilities of *p < 0.05

Despite the method to obtain the zein NPs, the pH of the surrounding matrix in which they are obtained is an important matter as it influences their integrity, quality, and ultimately their functionality. Zein is a protein from a natural source, and it possesses ideal properties to develop protein-based delivery carriers, however, zein NPs have the predisposition to aggregate and precipitate at neutral or higher pH values, since zein presents an isoelectric pH of 6.2 [42]. Therefore, to evaluate the potential application of MF-loaded zein NPs for oral delivery application, the release profile of MF from zein NPs was assessed by incubating the formulations for 2 h at 37 °C in a buffer that resembled the gastric environment (pH 1.2), followed by 6 h of incubation at 37 °C in a buffer that corresponds the intestinal environment (pH 6.8) (Fig. 3). To guarantee sink conditions, 1.5% (w/v) of Poloxamer 407 was added to both buffers. Afterward, the release profile of MF from zein NPs was compared with the free drug. The release pattern of free MF was approximately 40% in the gastric buffer after 15 min of experiment, followed by a release of 40% in intestinal buffer, reaching 80% of MF release, after 8 h of assay. Interestingly, the release of MF from zein NPs was found to be pH-dependent, demonstrating residual release of MF from zein NPs at gastric conditions followed by higher release values once incubated in the intestinal buffer. The release of MF from zein NPs in gastric buffer was similar for all the MF-loaded zein NPs formulations (5% MF 0.1 P, 5% MF 0.5P, and 10% MF 0.5P), with approximately 10% of MF release after 2 h of experiment. In contrast, when NPs were incubated in intestinal buffer, the 5% MF 0.5P formulation reached 40% of release after 150 min, followed by a plateau of up to 8 h. In comparison, the NPs 5% MF 0.1P and 10% MF 0.5P showed a release of 20% after 150 min, which was maintained until 8 h of the experiment. Overall, the release of MF from zein NPs was found to be pH-dependent since a higher release of MF from zein NPs was observed for all formulations when incubated in intestinal buffer (pH 6.8) contrarily to what was observed with the gastric buffer (pH 1.2). During the first 2 h of the experiment, MF molecules had poor diffusion from the NPs into the aqueous medium and after, under intestinal conditions, the release of MF increased that could mostly as a consequence of the erosion and/or relaxation of the NP matrix. Moreover, a detail that should be considered is the MF adsorption capacity to the NPs surface, instead of being loaded within the protein matrix, thus exposing the drug prematurely. This circumstance may be a consequence of low loading capacity of less than 10% for zein NPs, as previously reported [26, 48].

## Cytotoxicity of MF-loaded zein NPs

**Fig. 4:**
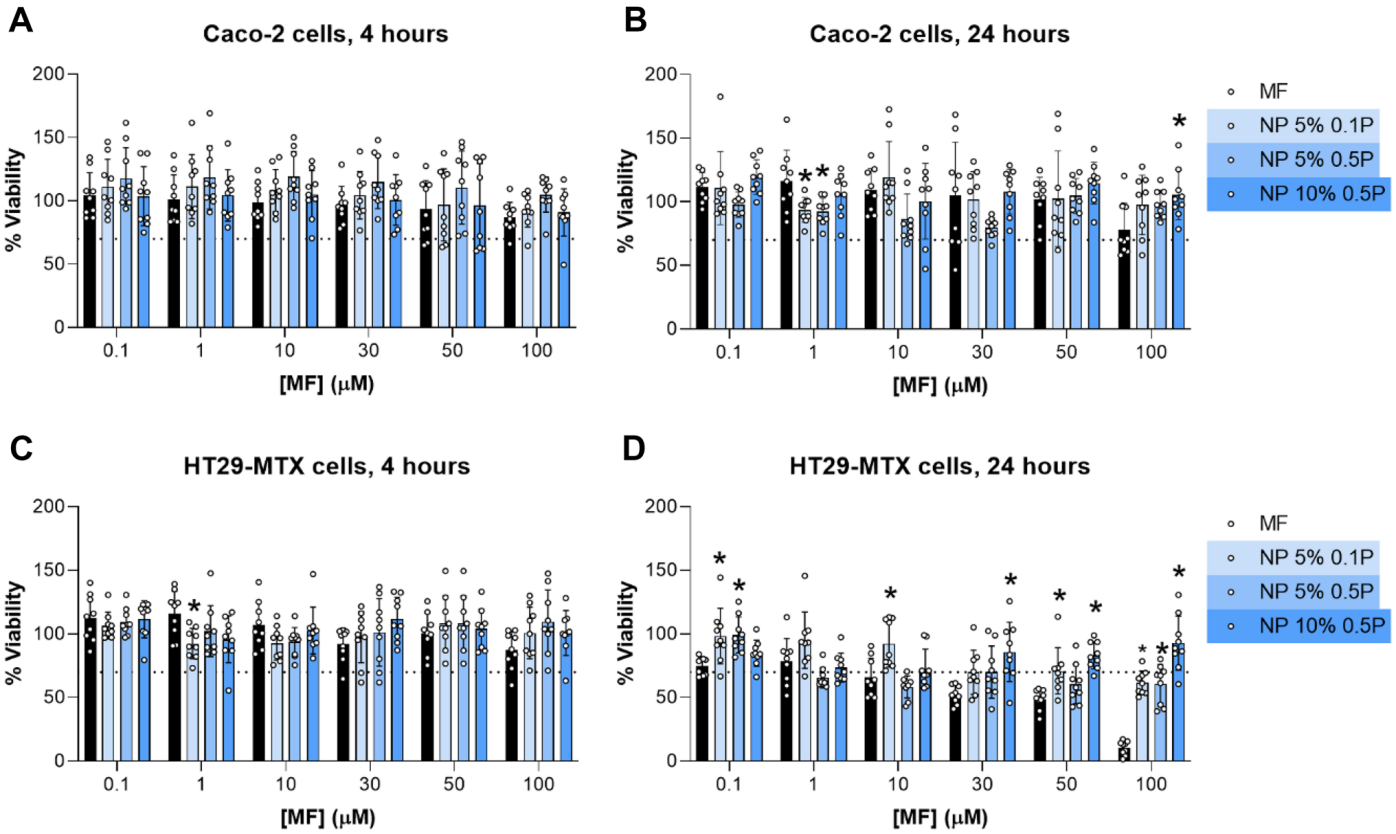
Cell viability of Caco-2 cells (**A**, **B**) and HT29-MTX cells (**C**, **D**), after 4 h and 24 h of incubation with different concentrations of free mometasone furoate (MF) and MF-loaded zein nanoparticles (NPs) at different theoretical loadings (TL) and different concentrations of Poloxamer 407: 5% or 10% MF-loaded zein NPs containing 0.1% (5% 0.1P) or 0.5% of Poloxamer 407 (5% 0.5P 10% 0.5P). Viability (%) was assessed using the triazolyl blue tetrazolium bromide (MTT) metabolic activity assay. Results are presented as mean ± SD (n = 3). One-way ANOVA followed by Tukey’s multiple comparison test were used for the statistical analyses. The significant differences were set at probabilities of *p < 0.05

The safety evaluation of a new formulation is a fundamental parameter to be addressed. The impact of MF-loaded zein NPs in the viability of Caco-2 cells (enterocyte-like cells) and HT29-MTX cells (mucin-producing cells) was assessed through the measurement of cellular metabolic activity, using the MTT colorimetric assay. Concentrations ranging from 0.1 to 100 μM (expressed as the amount of drug) of free MF and MF-loaded zein NPs were incubated with both cell lines for 4 h and 24 h. The results for cell viability are presented in Fig. 4. The zein protein is well recognized for its biocompatibility and low cytotoxicity profile, establishing it as a safe matrix suitable for numerous applications, including drug delivery [49]. Furthermore, free MF and MF-loaded zein NPs did not affect the viability of Caco-2 cells, after 4 h and 24 h of incubation (Fig. 4A, B) and HT29-MTX cells, after 4 h of incubation (Fig. 4C). The decrease in the viability of HT29-MTX cells caused by free MF after 24 h of incubation appears to be dose-dependent. In fact, zein NPs were able to decrease the apparent toxicity of MF after 24 h of incubation in HT29-MTX cells (Fig. 4D), allowing an increase of the CC_50_ values of the drug from around 30 μM to values higher than 100 μM. Overall, MF-loaded zein NPs appear to present the same toxicity profile as the free drug. Empty zein NPs within the concentration tested range, did not affect the viability of both colorectal cell lines (Suppl. Fig. 2), since viability values remained above the lower viability threshold of 70% [50].

## Permeability of MF-loaded zein NPs across colorectal cell monolayers

**Fig. 5:**
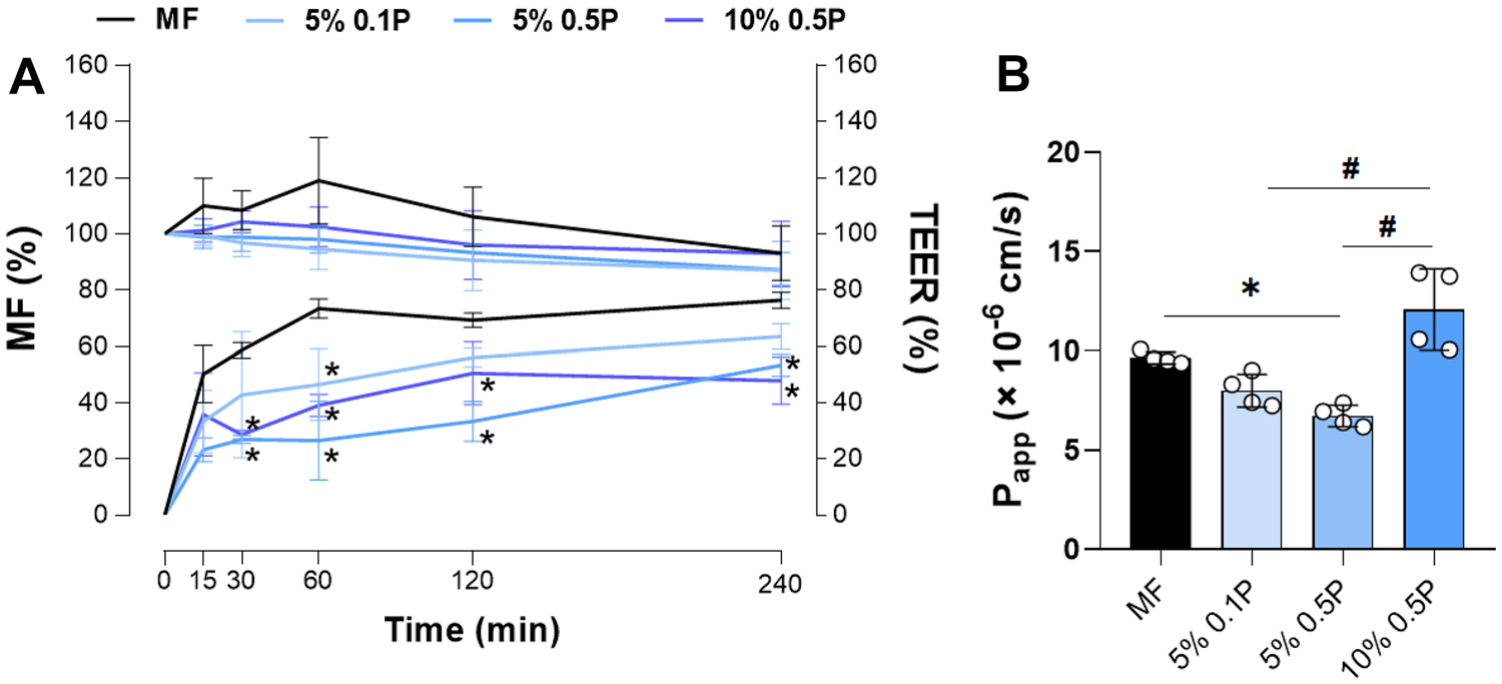
In vitro permeability studies. Cumulative permeability of MF (**A**) and apparent permeability coefficients (**B**) across Caco-2/HT29-MTX co-culture after incubation with free mometasone furoate (MF) (**A**) and MF-loaded zein nanoparticles (NPs) at different theoretical loadings (TL) and different concentrations of Poloxamer 407: 5% or 10% MF-loaded zein NPs containing 0.1% (5% 0.1P) or 0.5% of Poloxamer 407 (5% 0.5P 10% 0.5P), for 240 min

In this study, we used the widely accepted in vitro co-culture cell model, composed of Caco-2 and HT29-MTX to investigate the potential of MF-loaded zein NPs to permeate the intestinal epithelium. This model forms a functional epithelial barrier, suitable to determine the intestinal permeability, that comprises the two cell types that are most represented in the intestinal epithelium: enterocytes and mucus-producing goblet cells, obtained by the differentiation of Caco-2 cells and HT29-MTX cells, respectively, mirroring the physiological ratio of 9:1. Besides, it is described both cells that compose the monolayer are joined by tight junctions, mimicking the intestinal epithelial tissue, conferring more biological relevance to the model [51, 52]. The permeation of MF-loaded zein NPs was conducted from the apical to the basolateral side in Caco-2/HT29-MTX monolayers over 4 h. The data showed that the free MF diffuses rapidly across the Caco-2/HT29-MTX cell monolayer, with a permeability of around 80% and P_app_ of 9.6 × 10^−6^ cm.s^−1^ (Fig. 5). At the end of the experiment, the permeability of MF loaded into zein NPs was similar between 5% MF 0.5P NPs and 10% MF 0.5P NPs, reaching around 50% of permeability, while the 5% MF 0.1P formulation presented a slightly higher percentage or permeation, around 63%. Similar permeability profiles have been observed in other protein-based carriers [53]. Although we evaluated the permeability of MF up to 240 min, previous studies demonstrate that when orally administered, zein NPs remained in the gastrointestinal tract of rats for a period of at least 24 h post-administration [23, 48]. Additionally, release studies under simulated intestinal pH conditions also demonstrated the delay effect of zein matrix in the release kinetics of MF compared to free drug, which may also justify the overall decrease of MF permeated. Indeed, P_app_ values for zein NPs 5% MF 0.1P (8.0 ± 0.8 × 10^−6^ cm.s^−1^) and 5% MF 0.5P (6.9 ± 0.4 × 10^−6^ cm.s^−1^) were slightly lower compared to the free MF (9.6 ± 0.3 × 10^−6^ cm.s^−1^), while in the case of 10% MF 0.5P NPs, the P_app_ value (12.1 ± 0.4 × 10^−6^ cm.s^−1^) increased by 40% compared to free drug as a result of the higher drug loading content.

Commonly, the mucus layer that covers the surface of the intestinal epithelium represents one of the main barriers to NPs permeability [54]. The mucus is a complex hydrogel composed mainly by water and mucins and forms a viscoelastic network that limit the free diffusion of the compounds [55]. Specifically, NPs permeation across the mucus layer can be influenced by particle size, surface charge, and hydrophobicity [56]. The presence of mucus, produced by the goblet-like cells that compose the in vitro co-culture model used to assess the permeability, is likely responsible for the expected to hold up the path of NPs towards the epithelium surface. In addition, due to its hydrophobic character, MF may be capable to establish interactions with mucin formed in the cell model, as previously described by other hydrophobic molecules [23, 26, 57], reducing MF transport across intestinal monolayer [58], resulting in an underestimation of the permeability of such compound. Besides, zein demonstrated mucoadhesive properties, which can be translated in the increment of the residence time of the zein NPs in the gastrointestinal tract, and thus could favor the MF permeability at a higher extent, contributing to an improvement of its local and systemic bioavailability [18]. Thus, the mucoadhesive property may extend the time of MF in contact with the absorptive epithelial cells and could facilitate the establishment of a concentration gradient from the nanoparticulate matrix until the permeable membrane. Finally, the TEER values were maintained, indicating the integrity of the cell monolayer throughout the experiments (Fig. 5, right y-axis) [58]. This further reinforces the low cytotoxicity profile of zein NPs, as shown in the MTT metabolic activity assay.

The present study supports the hypothesis of zein being a promising carrier for oral delivery of hydrophobic drugs, namely MF, and open the possibility of reducing adverse effects caused by GCs administration. Indeed, zein NPs themselves can reduce hyperglycemia and improve glucose tolerance in rats [22], which are metabolic effects induced by GC treatment [1]. Furthermore, zein NPs are efficient nano-based systems capable of efficiently transport its cargo to the small intestine [58]. Once there, MF-loaded zein NPs is retained for longer periods in the gastrointestinal tract and can provide its sustained release, preventing the delivery of high doses of MF into the bloodstream, due to their mucoadhesive characteristics.

The present study opens several questions that will benefit from future research, such as a deeper insight into the pharmacokinetics profile of MF-loaded NPs and the analysis of long-term safety associated to MF-loaded zein NPs oral delivery. In any case, these NPs might constitute an alternative to develop effective strategies for preventing GC-induced metabolic adverse effects, such as hyperglycemia.

## Conclusion

In this work, we developed and characterized zein-based NP for the oral administration of MF. Zein NPs demonstrates 100 nm size range and narrow particle size distribution, conferring them potential for intestinal translocation. Nevertheless, permeability studies suggested that zein NPs retains MF transport across cell monolayers compared to the free drug, resulting in slow and gradual intestinal absorption. The lower MF release in gastrointestinal conditions can also support the differences in permeability kinetics. These results may lead to a more prolonged local anti-inflammatory effect and fewer adverse effects. The formulation developed in this study indicates successful carrying of MF to the intestine and future studies are welcome to investigate the use of MF-loaded zein NPs to treat intestinal inflammatory diseases.

### Supplementary Information

Below is the link to the electronic supplementary material.

Supplementary file1 (DOCX 45 KB)

Supplementary file2 (TIF 37 KB)

Supplementary file3 (TIF 32 KB)

## Data Availability

The collected and analyzed datasets during this study are available from the corresponding author upon reasonable request.
