# Supplementary figures and images for: Zein nanoparticles as oral carrier for mometasone furoate delivery
Source: Drug Deliv Transl Res. 2023 May 19;13(11):2948–59. doi: 10.1007/s13346-023-01367-y (PMC10545574; doi:10.1007/s13346-023-01367-y)

A

| **[MF] µg/mL** | **Abs (1)** | **Abs (2)** | **Abs 3 (3)** | **Mean** |
| --- | --- | --- | --- | --- |
| **0** | 0,019607 | 0,02456 | 0,030727 | 0,024965 |
| **5** | 0,19366 | 0,19163 | 0,18923 | 0,191507 |
| **10** | 0,37121 | 0,37343 | 0,3756 | 0,373413 |
| **20** | 0,89696 | 0,90087 | 0,90078 | 0,899537 |
| **40** | 1,8629 | 1,8705 | 1,8664 | 1,866600 |
| **60** | 2,4684 | 2,4444 | 2,4817 | 2,464833 |

B


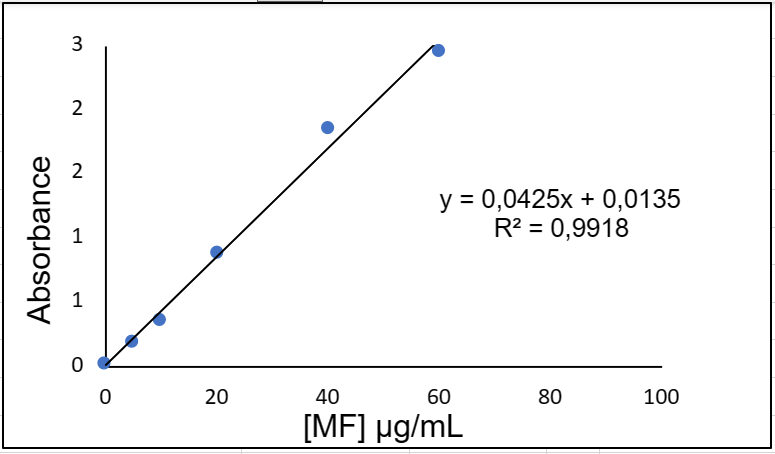


C

| **Amount of MF (µg/mL)** | **% drug content** | **% RSD** |
| --- | --- | --- |
| 40 | 101.5 | 0.758 |
| 101.3 |
| 99.8 |

Inter-day data, n = 3 replicates

Supplement: Supplementary file 1 — Supplementary file1 (DOCX 45 KB) [file 13346_2023_1367_MOESM1_ESM.docx]

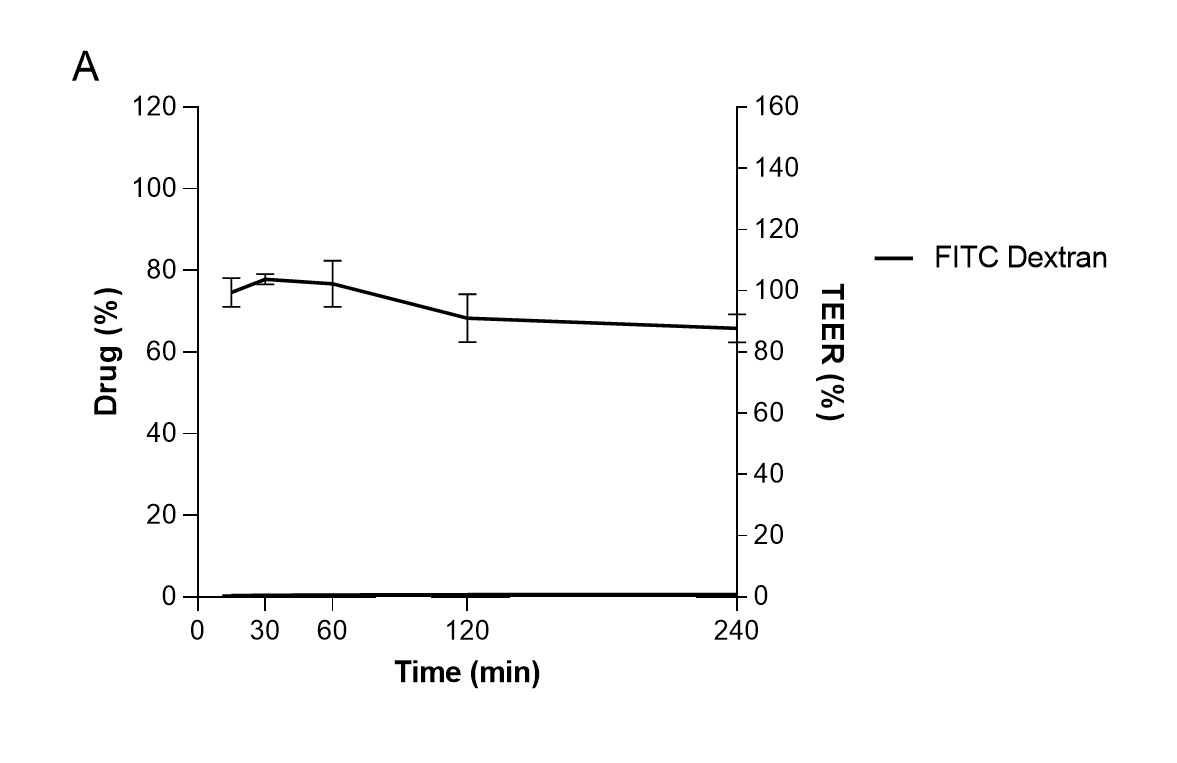

Supplement: Supplementary file 2 — Supplementary file2 (TIF 37 KB) [file 13346_2023_1367_MOESM2_ESM.tif]

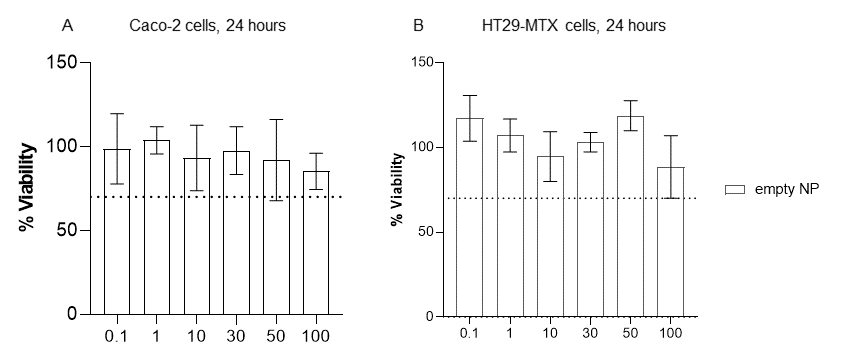

Supplement: Supplementary file 3 — Supplementary file3 (TIF 32 KB) [file 13346_2023_1367_MOESM3_ESM.tif]
